# Emergent Quantum Mechanics: David Bohm Centennial Perspectives

**DOI:** 10.3390/e21020113

**Published:** 2019-01-26

**Authors:** Jan Walleczek, Gerhard Grössing, Paavo Pylkkänen, Basil Hiley

**Affiliations:** 1 Phenoscience Laboratories, Novalisstrasse 11, Aufgang F, 10115 Berlin, Germany; 2 Austrian Institute for Nonlinear Studies, Akademiehof, Friedrichstrasse 10, 1010 Vienna, Austria; 3 Department of Philosophy, History, and Art Studies, P.O. Box 24 (Unioninkatu 40 A), University of Helsinki, FI-00014 Helsinki, Finland; 4 Department of Cognitive Neuroscience and Philosophy, University of Skövde, P.O. Box 408, SE-54128 Skövde, Sweden; 5 Department of Physics and Astronomy, University College London, Gower Street, London WC1E 6BT, UK

**Keywords:** quantum ontology, nonlocality, time-symmetry, retrocausality, quantum causality, conscious agent, emergent quantum mechanics, Bohmian mechanics, de Broglie-Bohm theory

## Abstract

Emergent quantum mechanics (EmQM) explores the possibility of an ontology for quantum mechanics. The resurgence of interest in realist approaches to quantum mechanics challenges the standard textbook view, which represents an operationalist approach. The possibility of an ontological, i.e., realist, quantum mechanics was first introduced with the original de Broglie–Bohm theory, which has also been developed in another context as Bohmian mechanics. This Editorial introduces a Special Issue featuring contributions which were invited as part of the David Bohm Centennial symposium of the EmQM conference series (www.emqm17.org). Questions directing the EmQM research agenda are: Is reality intrinsically random or fundamentally interconnected? Is the universe local or nonlocal? Might a radically new conception of reality include a form of quantum causality or quantum ontology? What is the role of the experimenter agent in ontological quantum mechanics? The Special Issue also includes research examining ontological propositions that are not based on the Bohm-type nonlocality. These include, for example, local, yet time-symmetric, ontologies, such as quantum models based upon retrocausality. This Editorial provides topical overviews of thirty-one contributions which are organized into seven categories to provide orientation.

“Towards Ontology of Quantum Mechanics and the Conscious Agent” was the heading of the David Bohm Centennial symposium as part of the Emergent Quantum Mechanics (EmQM) conference series (www.emqm17.org). The three-day symposium was held at the University of London, right next to Birkbeck College, the final academic home of David Bohm. The symposium offered an open forum for critically evaluating the prospects and significance—for 21st century physics—of ontological quantum mechanics, an approach which David Bohm helped pioneer. The Editorial introduces contributions featuring the original research of the EmQM symposium speakers, as well as additional researchers who are exploring the ontological implications of quantum mechanics. The contributions are thematically organized as follows: (1) Quantum Ontology and Foundational Principles, (2) The Continuing Impact of the Bohmian Theory, (3) Beyond the Bohmian Theory: New Developments, (4) Quantum Ontology and Time: Retrocausality and Irreversibility, (5) Entropy, Thermodynamics, and Emergent Quantum Gravity, (6) Alternative Quantum Models and Tools, and (7) Advanced Quantum Experimentation. 

## 1. Quantum Ontology and Foundational Principles

Foundational principles and concepts are introduced to help guide thinking about the validity of ontological propositions for quantum mechanics. Tim Maudlin starts with the key insight that any possible ontology for quantum mechanics necessitates “a radical change in our understanding of physical ontology” [1]. Employing as an example the Aharonov-Bohm effect, he develops a method for “clarifying what the commitments of a clearly formulated physical theory are”. Referring to the well-known conceptual challenges in interpreting quantum theory, Maudlin concludes by noting that if “physicists were to adopt this method… to convey physical theories clearly and unambiguously, many conceptual problems could be avoided”.

Jan Walleczek advances the concept of agent inaccessibility as a fundamental principle in quantum mechanics, based on the objective uncomputability of quantum processes as a formal limit [2]. In support of the Bohmian theory, the proposal of an agent-inaccessibility principle presents an alternative position to the standard textbook view of quantum indeterminism. Walleczek concludes that the 20th century quantum revolution need not imply “a radical shift from determinism to indeterminism” but that—based on current knowledge—it is only valid to assert that “the quantum revolution signifies the profound discovery of an agent-inaccessible regime of the physical universe”.

Maurice De Gosson next introduces the mathematics of Poincare’s recurrence theorem, and the associated notion of ‘superrecurrence’, in relation to the properties of symplectic topology, as applied to quantum mechanics [3]. De Gosson suggests that these recurrence properties “are closely related to Emergent Quantum Mechanics since they belong to the twilight zone between classical (Hamiltonian) mechanics and its quantization”, and he views these properties “as *imprints of the quantum world* on classical mechanics in its Hamiltonian formulation”.

William Seager provides a 21st century interpretation of the philosophy and scientific metaphysics of David Bohm [4]. Specifically, Seager examines three core features of Bohm’s foundational views, namely “the holistic nature of the world, the role of a unique kind of information as the ontological basis of the world, and the integration of mentality into this basis as an essential and irreducible aspect of it”. Importantly, Seager corrects the persistent, but flawed, view that Bohmian ontology “is a return to a classical picture of the world“, and he explains that “Bohm’s metaphysics is about as far from that of the Newtonian classical metaphysical picture of the world as one could get”.

## 2. The Continuing Impact of the Bohmian Theory

The focus of the second category is the continuing impact, based on recent assessments and conceptual innovations, of the original de Broglie–Bohm (dBB) theory and Bohmian mechanics. The opening article by Basil Hiley and Peter Van Reeth engages the historically controversial problem of the reality of Bohmian quantum trajectories [5]. The authors argue that the previous “conclusion that the Bohm trajectories should be called ‘surreal’… is based on a false argument.” Specifically, Hiley and Van Reeth show that “standard quantum mechanics produces exactly the same behavior as the Bohmian approach so it cannot be used to conclude the Bohm trajectories are ‘surreal’.”

Robert Flack and Basil Hiley—again addressing the problem of quantum trajectories—explore “the relationship between Dirac’s ideas, Feynman paths, and the Bohm approach” [6]. After studying the relationship in detail, Flack and Hiley propose that “a Bohm ‘trajectory’ is the average of an ensemble of actual individual stochastic Feynman paths”, and that, therefore, these paths “can be interpreted as the mean momentum flow of a set of individual quantum processes and not the path of an individual particle.”

Nicolas Gisin, next, clarifies the long-standing debate between those in the mainstream of physics who argue that the Bohmian approach is “disproved by experiments”, and those who insist that “Bohmian mechanics makes the same predictions as standard quantum mechanics” [7]. After performing a careful analysis, Gisin arrives at the conclusion that “…Bohmian mechanics is deeply consistent”, and he notes that “Bohmian mechanics… could inspire brave new ideas that challenge quantum physics.”

Dustin Lazarovici, Andrea Oldofredi, and Michael Esfeld continue with key arguments in support of the physical consistency of Bohmian mechanics [8]. In particular, these authors offer a critical assessment of standard no-hidden-variables theorems, which have long been used to challenge the plausibility of the Bohmian ontology. In particular, they argue that “far from challenging—or even refuting—Bohm’s quantum theory, the no-hidden-variables theorems, in fact, support the Bohmian ontology for quantum mechanics.”

Oliver Passon, next, tackles a common misconception regarding the dBB theory [9], namely the specific criticism that the theory “not only assigns a position to each quantum object but also contains the momenta as ‘hidden variables’.” In response to this perceived inconsistency, he points out that the measurement of momentum in the dBB theory is strictly contextual and does not reveal a “preexisting value”, and that, therefore, the Bohmian interpretation “is not only a consistent interpretation of quantum mechanics but includes also ‘quantum weirdness’—like any other interpretation of quantum theory.”

Travis Norsen offers an explanation of the Born-rule statistics for the dBB pilot-wave theory [10]. In the task of finding a realist account of the Born rule expressing the probability distribution of measurement outcomes, Norsen compares the two competing approaches from the literature and he finds that “there is somewhat less conflict between the two approaches than existing polemics might suggest, and that indeed elements from both arguments may be combined to provide a unified and fully-compelling explanation, from the postulated dynamical first principles, of the Born rule.”

Ángel Sanz highlights the impact of Bohmian theory—beyond mere theoretical significance, namely, as a “useful resource for computational and interpretive purposes in a wide variety of practical problems” [11]. Specifically, an analysis of “the problem of the diffraction of helium atoms from a substrate consisting of a defect with axial symmetry on top of a flat surface” is performed, and the behavior of Fermatian trajectories (optical rays), Newtonian trajectories, and Bohmian trajectories is compared, whereby, the latter are shown to “behave quite differently, due to their implicit non-classicality”.

The final article in this category is contributed by Roderich Tumulka [12]. He provides an overview of Bohmian mechanics, and then continues to describe “more recent developments and extensions of Bohmian mechanics, concerning, in particular, relativistic space–time and particle creation and annihilation.” Tumulka concludes by emphasizing that the described theoretical work represents “the most plausible ontological theory of quantum mechanics in relativistic space–time”, and it, therefore, holds great promise “as a fully satisfactory extension of Bohmian mechanics, to relativistic space–time.”

## 3. Beyond the Bohmian Theory: New Developments

This category features research pursuing ideas beyond the Bohmian theory and its typical interpretation. Although the researchers agree that the notion of nonlocality is essential to an ontological quantum mechanics, new developments are explored, based on the assumptions and propositions that are not normally covered by the dBB theory and by Bohmian mechanics.

Gerhard Grössing, Siegfried Fussy, Johannes Mesa Pascasio, and Herbert Schwabl present a model of quantum reality that “does not need wave functions”, and one that assumes a “cosmological solution” to the problem of nonlocality [13]. That is, the researchers propose “that from the beginning of the universe, each point in space has been the location of a scalar field representing a zero-point vacuum energy that nonlocally vibrates at a vast range of different frequencies, across the whole universe.” Assuming this cosmological nonlocality, the authors provide classical computer simulations of double- and n-slit interference patterns, which reveal trajectories that “are in full accordance with those obtained from the Bohmian approach.”

Mohamed Hatifi, Ralph Willox, Samuel Colin, and Thomas Durt present an analysis of a quantum model inspired by “the properties of bouncing oil droplets”—as observed by the so-called ‘walkers’ in non-equilibrium experiments—and which “have attracted much attention because they are thought to offer a gateway to a better understanding of quantum behavior” [14]. In particular, the authors perform an analysis comparing “walker phenomenology in terms of the de Broglie–Bohm dynamics and of a stochastic version, thereof.” They conclude that “the programs that aim at simulating droplet dynamics with quantum tools or at describing the emergence of quantum dynamics, based on droplet dynamics…raise challenging fundamental questions.”

Mojtaba Ghadimi, Michael Hall, and Howard Wiseman describe research findings related to the Many Interacting Worlds (MIW) proposal, which is “a new approach to quantum mechanics, inspired by Bohmian mechanics” [15]. The MIW proposal represents an entirely novel way of addressing the problem of nonlocality in Bohmian mechanics, and while “it is conceptually clear how the interaction between worlds can enable this strong nonlocality”, a proof by simulation has not been possible so far. In the present contribution, the authors now “report significant progress in tackling one of the most basic difficulties that needs to be overcome: Correctly modelling wave functions with nodes.”

## 4. Quantum Ontology and Time: Retrocausality and Irreversibility

Time-related aspects and interpretations of quantum mechanics are the focus of this category. The first three articles present work that considers, in three distinct ways, the possible relationships between the implicit time-symmetry of the quantum formalism and physical ontology. The final article discusses the concept of fundamental irreversibility in nature. 

Emily Adlam starts by noticing that “the physics community has come to take seriously, the possibility that the universe might contain physical processes which are spatially nonlocal, but there has been no such revolution with regard to the possibility of temporally nonlocal processes” [16]. The author suggests that “the assumption of temporal locality is actively limiting progress in the field of quantum foundations”, and then offers an investigation into “the origins of the assumption, arguing that it has arisen for historical and pragmatic reasons rather than good scientific ones.” Adlam concludes with the proposal that “once we accept that the universe may be generically nonlocal, across both time and space, it becomes at least plausible that quantum theory as we know it is simply the local limit of a global theory, which applies constraints across the whole of space and time.”

Kenneth Wharton introduces a new class of retrocausal models that he hopes will “guide further research into space–time-based accounts of weak values, entanglement, and other quantum phenomena” [17]. This work is inspired by the recognition that “globally-constrained classical fields provide an unexplored framework for modeling quantum phenomena, including apparent particle-like behavior.” In relation to prior retrocausal models in the literature, Wharton explains that “the central novelties in the class of models discussed here are: (1) Using fields (exclusively) rather than particles; and (2) introducing uncertainty to even the initial and final boundary constraints.”

Nathan Argaman reconsiders a central tenet of Bell’s nonlocality theorem—the causal arrow of time. He points out that “the physical assumptions regarding causality are seldom studied in this context, and often even go unmentioned, in stark contrast with the many different possible locality conditions which have been studied and elaborated upon” [18]. Argaman envisions the future generalization of “retrocausal toy-models to a full theory—a reformulation of quantum mechanics—in which the standard causal arrow of time would be replaced by a more lenient one: An arrow of time applicable only to macroscopically-available information.” He concludes by suggesting that for “such a reformulation, one finds that many of the perplexing features of quantum mechanics could arise naturally, especially in the context of stochastic theories.”

Lajos Diósi compares two fundamental concepts of irreversibility which, as he emphasizes in this work, have “emerged and evolved with few or even no interactions” [19]. First, the concept of universal gravity-related irreversibility, and, second, irreversibility in “quantum state reductions, unrelated to gravity or relativity but related to measurement devices”. The author first summarizes the two concepts and then highlights the significant fact that the precise relationship “between the Planckian and the Schrödinger–Newton unpredictability of our space–time” remains unknown. In conclusion, Diósi notes that “Planckian unpredictability survives non-relativistically—for massive macroscopic quantized degrees of freedom.”

## 5. Entropy, Thermodynamics, and Emergent Quantum Gravity

Theoretical issues are addressed linking entropic and thermodynamical considerations with quantum systems and possible emergent quantum phenomena. The first article by Jen-Tsung Hsiang and Bei-Lok Hu starts out by explaining that–in view of “quantum mechanics as an emergent theory”—thermodynamical theory “is perhaps one of the most powerful theories and best understood examples of emergence in physical sciences, which can be used for understanding the characteristics and mechanisms of emergent processes” [20]. The authors stress that even for the initial goal of developing a viable “quantum thermodynamics”, there are “many new issues which need be addressed and new rules formulated.” For the present contribution, Hsiang and Hu offer “quantum formulations of equilibrium thermodynamic functions and their relations for Jarzynski’s classical thermodynamics at strong coupling”.

Osvaldo Civitarese and Manuel Gadella start with a review of “the concept of entropy in connection with the description of quantum unstable systems”, whereby the goal of this work is to show “that a comprehensive scheme leading to the definition of entropy for resonances can be rigorously designed by adopting path integration techniques” [21]. Specifically, these authors advance “a proper definition of this entropy based on the use of Gamow states as state vectors for resonances.” In conclusion, Civitarese and Gadella explain that the “resulting entropy is complex, with an imaginary part which gives an account for the interactions of decaying states with their surroundings.”

Arno Keppens pursues a “complex systems approach, as a kind of toy model, for identifying space–time’s ontological micro-constituents and their interaction, i.e., their sub-quantum dynamics” [22]. Towards that end, he combines two research strategies, whereby, the first views “gravity as an entropic phenomenon”, and the second derives “a sub-quantum interaction law” from the solution of Einstein’s field equations. Keppens argues that “novel views on entropic gravity theory result from this approach, which eventually provides a different view on quantum gravity and its unification with the fundamental forces.”

Massimo Tessarotto and Claudio Cremaschini formulate a Bohmian trajectory-based representation for the quantum theory of the gravitational field [23]. Specifically, the researchers describe “the basic principles of a new trajectory-based approach to the manifestly-covariant quantum gravity (CQG) theory.” Importantly, their work provides “new physical insight into the nature and behavior of the manifestly-covariant quantum-wave equation and corresponding equivalent set of quantum hydrodynamic equations that are realized by means of CQG-theory.” Remarkably, as Tessarotto and Cremaschini emphasize, based on their approach “the existence of an emergent gravity phenomenon is proven to hold.”

## 6. Alternative Quantum Models and Tools

A wide variety of different approaches, including those proposing the construction of alternative ontologies, are grouped together in this category. The five contributions range from quantum models that seek to explain quantum phenomena by local, yet unconventional, accounts of physical reality to a quantum model based on an observer-independent event ontology.

Tim Palmer presents a cosmological model in “which the universe evolves deterministically and causally, and from which space–time and the laws of physics in space–time are emergent” [24]. Significantly, the author counters the view that a Bohm-type nonlocality—in view of Einstein–Podolsky–Rosen (EPR)-type quantum-entanglement correlations—might exist in reality. The model “challenges the conclusion that the Bell Inequality has been shown to have been violated experimentally, even approximately”, and it “postulates the primacy of a fractal-like ‘invariant set’ geometry”. Palmer concludes by discussing the relationships between the Invariant Set Theory, which is “deterministic and locally causal”, and the Bohmian theory, the cellular automaton interpretation of quantum theory and the p-adic quantum theory.

Thomas Filk continues the challenge for the need of a Bohm-type, nonlocal ontology as an explanation of the EPR-type quantum-entanglement correlations [25]. In particular, as an alternative, he describes “an interpretation of the mathematical formalism of standard quantum mechanics in terms of relations”, and from this he develops “the notion of a relational space.” In this description, as the author explains, “entanglement is interpreted as a relation between two entities (particles or properties of particles).” Importantly, in the proposed relational view, “the concept of ‘locality’ receives a completely different meaning when the positions or locations of entities (objects or events) are defined in a relational sense, as compared to an absolute space or space–time.” In conclusion, Filk discusses the quantum measurement problem, from the perspective of this relational interpretation. 

Dimiter Prodanov’s contribution describes “the mathematical foundations of the scale relativity theory, its link to stochastic mechanics, and the theory of the Burgers equation” [26]. This work is motivated “by the premise that inherently nonlinear phenomena need development of novel mathematical tools for their description.” In particular, Prodanov investigates “the potential of stochastic methods for simulations of quantum–mechanical and convection–diffusive systems”, whereby, the “presented numerical approaches can be used… for simulations of nanoparticles or quantum dots, which are mesoscopic objects and are expected to have properties intermediate between macroscopic and quantum systems”.

Louis Kauffman reviews “previous results about discrete physics and non-commutative worlds” [27]. As the author points out, important “aspects of gauge theory, Hamiltonian mechanics, relativity and quantum mechanics arise naturally in the mathematics of a non-commutative framework for calculus and differential geometry.” The article explores “the structure and consequences of constraints linking classical calculus and discrete calculus formulated via commutators.” Specifically for the reported second-order constraint, which is “based on interlacing the commutative and non-commutative worlds”—as Kauffman reports—“leads to an equivalent tensor equation at the pole of geodesic coordinates for general relativity”.

Rodolfo Gambini and Jorge Pullin provide a short review of the Montevideo interpretation of quantum mechanics [28]. Briefly, in their account of quantum phenomena, Gambini and Pullin “adopt an interpretation that provides an objective criterion for the occurrence of events”, whereby, for that purpose they are constructing “an ontology of objects and events”. Notably, in this alternative to the more familiar realist interpretations, the quantum events represent “actual entities” which are independent of any observers. Importantly, the Montevideo interpretation “is formulated entirely in terms of quantum concepts, without the need to invoke a classical world.”

## 7. Advanced Quantum Experimentation

The final category is devoted to experiments, and their interpretation, targeting advanced research questions in quantum foundations, as well as practical applications. The first article is by Lukas Mairhofer, Sandra Eibenberger, Armin Shayeghi, and Markus Arndt, who present quantum-interference experiments with biomolecules, and discuss the sensitivity to weak magnetic fields of the observed fringe patterns [29]. Under suitable conditions, “the molecules can…be prepared in superpositions of position and momentum”, the authors write, “even though we can assign classical attributes such as internal temperatures, polarizabilities, dipole moments, magnetic susceptibilities and so forth to them”. The researchers go on to explain that “macromolecular interferometry has very practical applications in metrology, for the measurement of electronic, optical, and even magnetic molecular properties.” Specifically, the authors report data for “quantum interference of the pre-vitamin 7-dehydrocholesterol”, and present the key finding that “even very small magnetic contributions can become accessible in matter-wave assisted deflectometry.”

Lev Vaidman and Izumi Tsutsui offer a conceptual analysis of “the history of photons in a nested Mach–Zehnder interferometer with an inserted Dove prism” [30]. The analysis refers to previous experimental results which “became the topic of a very large controversy”, as the authors explain. This contribution by Vaidman and Tsutsui serves to clarify the involved issues. Included in the article is an analysis also of the “nested interferometer in the framework of the Bohmian interpretation of quantum mechanics.”

Finally, Robert Flack, Vincenzo Monachello, Basil Hiley, and Peter Barker describe a method for measuring the weak value of spin, for atoms using a variant of the original Stern–Gerlach apparatus [31]. The purpose of the methodological design is to enable the testing of “the original Bohm approach”, which must use “non-relativistic atoms”. Specifically, the described experiment “is designed to measure the real part of the weak value of spin for an atomic system”, in this case for helium atoms. Reported in this work is a “full simulation of an experiment for observing the real part of the weak value”. The obtained results suggest that a “displacement of the beam of helium atoms in the metastable 2^3^S_1_ state…is within the resolution of conventional microchannel plate detectors indicating that this type of experiment is feasible.”

## 8. Outlook

The wide range of perspectives which were contributed to this Special Issue on the occasion of David Bohm’s centennial celebration, provide ample evidence for the continuing possibility of an ontological quantum mechanics. In fact, the case for realist approaches towards explaining quantum phenomena, including in the account of EPR-type quantum correlations, has only strengthened, in recent years. Pivotal to this emerging development—for which stands the project of emergent quantum mechanics or EmQM—has been the following realization: A physical ontology for the quantum level represents a measurement-dependent, contextual, or relational ontology; that is, the advancement of ‘quantum ontology’, as a scientific concept, marks a clear break with classical ontological propositions in the form of direct or naïve realism. Indeed, such an approach to ontology is a vital part of David Bohm’s legacy. He noted that, in classical ontological theories in physics, there has been a tendency to assume that the basic concepts of the theory correspond to independently existing realities, i.e., to realities that are not dependent either on context or deeper levels of being. By contrast, in his ontological interpretation of quantum theory, the basic concepts, such as “particle” or “momentum”, reflect a reality that is inherently dependent either on context, or on deeper levels, or on both. For the future, instead of denying the possibility of a ‘quantum reality’, the mainstream of quantum physics might embrace, and join in, the search for unconventional causal structures and non-classical ontologies, which can be fully consistent with the known record of quantum observations in the laboratory.
